# HSPB6: A Potential Prognostic Biomarker, Inhibiting the Epithelial–Mesenchymal Transition (EMT) Process Through the PI3K/Akt Signaling Pathway Based on the Machine Learning and Experimental Validation

**DOI:** 10.1155/humu/4843618

**Published:** 2026-04-20

**Authors:** Jian-she Wang, Yi-fan Qiu, Lu Zhang, Bo Ji, Sen Liang, Ya-Xuan Wang, Hai-xia Zhu

**Affiliations:** ^1^ Department of Urology, Yinfeng (Jinan) Hospital, Jinan, Shandong, China; ^2^ Department of Urology, First Affiliated Hospital of Harbin Medical University, Harbin, Heilongjiang, China, hrbmu.edu.cn; ^3^ Jiangsu Province Geriatric Hospital, Nanjing, Jiangsu, China; ^4^ Department of Urology, Fourth Affiliated Hospital of Guangxi Medical University, Liuzhou, China; ^5^ Cancer Research Center Nantong, Affiliated Tumor Hospital of Nantong University and Nantong Tumor Hospital, Nantong, Jiangsu, China

**Keywords:** biomarker, bladder cancer, epithelial–mesenchymal transition, HSPB6, prognostic model

## Abstract

Bladder cancer (BC) is a prevalent malignant tumor worldwide, posing a significant public health burden and challenge to human society. Current therapeutic modalities for BC include surgical treatment, radiotherapy, chemotherapy, targeted therapy, and immunosuppressive therapy. However, almost all patients experience disease progression and ultimately succumb to BC. Our study demonstrated that elevated expression of Heat Shock Protein Beta‐6 (HSPB6) correlated with higher clinical grades and stages, establishing it as an independent prognostic risk factor for BC. Enrichment analysis indicated that HSPB6 is associated with the extracellular matrix in BC. Experimental validation revealed that HSPB6 overexpression inhibits the proliferation of BC cell line T24. This effect may be achieved by inhibiting the PI3K/Akt signaling pathway, which in turn leads to inhibition of epithelial–mesenchymal transition (EMT). Furthermore, we developed a prognostic risk model that incorporated DDR2, DPYSL3, MFAP5, PDGFRB, and SPOCD1, allowing accurate prediction of patient outcomes based on immunological status. In conclusion, this study highlights that HSPB6 overexpression can restrain the proliferation of BC cells and inhibit EMT, underscoring its potential as a diagnostic marker and therapeutic target in BC.

## 1. Introduction

Bladder cancer (BC) is a malignant tumor that affects the inner lining of the bladder and typically originates from the epithelial cells of the bladder. Currently, it is one of the most common cancers worldwide, with a significantly higher incidence in men than in women [1, 2]. The incidence of BC gradually increases with age, with higher rates observed in individuals aged > 60 years. Certain regions such as North America and Europe have higher rates of BC, whereas some developing countries have reported relatively lower rates. Environmental factors, lifestyle choices, and genetic background may contribute to these differences [3, 4]. Smoking is the primary risk factor for BC, with smokers having a significantly higher risk of developing the disease than nonsmokers [5, 6]. Long‐term exposure to certain chemicals, such as aromatic amines and anilines, is also associated with the occurrence of BC, particularly in specific industrial environments [7–9]. Early symptoms of BC include hematuria (blood in the urine), frequent urination, urgency, and painful urination. Diagnostic methods include symptom assessment, imaging studies (such as ultrasound, CT, and MRI), and cystoscopy, which allow direct observation of the bladder lining and biopsy to confirm the presence of cancer cells [10, 11]. Treatment options include surgical intervention, pharmacological therapy (such as chemotherapy and immunotherapy), and radiation therapy. Chemotherapy may be administered before or after surgery to reduce the risk of tumor recurrence, whereas immunotherapy (such as the BCG) is suitable for certain types of BC, activating the body′s immune system to combat cancer [12–14]. With advancements in medicine, new treatment methods and technologies continue to emerge, providing patients with more treatment options. An increasing number of studies have revealed that certain genes play a crucial role in specific types of tumors [15, 16].

The heat shock protein (HSP) family comprises proteins that participate in reaction to various cellular stresses, including heat stress, oxidative stress, and exposure to chemical agents [17]. This family is divided into several subfamilies including HSP60, HSP70, HSP90, and small heat shock proteins (sHSPs) [18, 19]. Heat Shock Protein Beta‐6 (HSPB6) is widely expressed in tissues, such as the heart, skeletal muscle, and nervous system [20–22]. Its main functions include cellular protection, maintenance of protein homeostasis, and regulation of apoptosis. The role of HSPB6 in cancer has received increasing attention, as its expression is upregulated in various tumors. HSPB6 plays various roles in different cancers, including promotion of lung cancer progression [23] and chemotherapy resistance in ovarian cancer [24]. HSPB6 is also considered a potential biomarker because its expression level is correlated with the prognosis of certain cancers and may be used to assess patient response to treatment [25, 26]. Our study further explored the specific mechanisms of HSPB6 and its potential applications in BC.

Epithelial–mesenchymal transition (EMT) is a transformative process where epithelial cells lose their epithelial characteristics, such as cell polarity and cell junctions, while gaining mesenchymal cell properties, including cell motility and matrix‐degrading capacity. It plays an extremely critical role in the initiation and progression of various tumors including BC, and serves as a key process for tumor cells to acquire invasiveness, metastatic potential, and drug resistance [27]. Owing to the pivotal role of EMT in tumor progression, therapeutic strategies targeting EMT have become a research focus. Current studies mainly concentrate on blocking the EMT process or reversing the EMT state, so as to inhibit the metastasis and drug resistance of tumor cells. These strategies include inhibiting N‐cadherin and activating the expression of E‐cadherin, among others [28, 29]. Numerous studies have indicated that members of the HSP family are closely associated with the EMT process in tumors. Therefore, this study will focus on investigating the relationship between HSPB6 and EMT in BC [30, 31].

## 2. Material and Methods

### 2.1. Data Acquisition

In this study, we collected transcriptome data and clinical information for 394 tumor cases and 87 normal cases from six Gene Expression Omnibus (GEO) cohorts, specifically GSE13507, GSE3167, GSE52519, and GSE65635, all accessed through the GEO database. Furthermore, we acquired transcriptome data and associated clinical details for 431 cases from the TCGA database, which contain 412 BC patients and 19 normal individuals. The clinical data of GSE13507 and TCGA_BLCA are attached in Tables S1 and S2.

### 2.2. Enrichment Analysis

We first employed Gene Ontology (GO) analysis, using the “clusterProfiler” package to functionally annotate differentially expressed genes. The GO analysis identified enrichments in three areas by comparing genes with relevant GO terms. Subsequently, using the “enrichKEGG” function from the “clusterProfiler” package, we compared our gene list with pathways in the KEGG database, calculating the enrichment *p* values for each pathway and applying multiple testing corrections.

### 2.3. Identification of BC‐Specific Genes

We employed the weighted gene coexpression network analysis (WGCNA) package to examine the genetic landscape of BC. The modules established a framework for prioritizing genes that may be associated with disease progression and prognosis. We utilized advanced machine learning techniques to enhance the selection of key genes. We utilized the e1071 package to conduct support vector machine recursive feature elimination (SVM‐RFE), a method for feature selection that systematically eliminates the least significant features to pinpoint the most pertinent genes. Concurrently, we performed random forest analyses utilizing the randomForest package, capitalizing on its strong feature‐ranking abilities to refine the selection of candidate genes.

We conducted friend analyses using the GOSemSim package to assess semantic similarities among genes based on their associated GO terms. This method yielded enhanced understanding of functional relationships and biological significance among the chosen genes. We performed survival analysis utilizing Cox regression, executed through the survival package.

### 2.4. Survival Analysis

This study utilized R to perform survival analysis to evaluate factors affecting patient survival time. We primarily used the “survival” and “survminer” packages for data analysis and visualization, respectively. First, we created a survival object using the “Surv” function, which included survival time and event status. To assess the impact of various factors on survival time, we constructed a Cox proportional hazards model using the “coxph” function. The validity of the model was verified through a residual analysis and tests of the proportional hazards assumption. Finally, we used the “ggsurvplot” function to plot the survival curves, visually demonstrating the survival differences among groups. These results provide important insights into key factors influencing patient survival.

### 2.5. Development of Prognostic Model Using Machine Learning

To construct a highly accurate and consistent prognostic model, we integrated several machine learning algorithms into various combinations. The algorithms employed included Enet, GBM, glmBoost, plsRglm, random forest, Ridge, Stepglm, and XGBoost. We assessed each algorithmic combination based on the area under the curve (AUC) values.

### 2.6. Transfection Assay

Electroporation was performed using the Neon Transfection System according to the manufacturer′s instructions. Healthy logarithmically growing target cells were harvested by digestion and centrifugation to remove the supernatant (suspended cells were directly collected). The cells were then resuspended in PBS for counting, and 1 × 10^6^ cells were transferred into an EP tube, followed by centrifugation to obtain a cell pellet. The cells were resuspended in electroporation buffer, and the plasmid was added and mixed thoroughly. The optimal electroporation parameters were applied, and the prepared mixture was electroporated along with an EGFP control group. Electroporated cells from each group were transferred to the corresponding culture dishes and placed in an incubator. After 24 h, the cell status was observed, and the electroporation efficiency was assessed based on the EGFP control group. After 48 h of cell culture, the corresponding selection drug was used to screen electroporated target cells.

### 2.7. Wound Healing Assay

Initially, cells are plated onto a six‐well plate at a suitable density to promote the formation of a confluent monolayer within 24 h. A straight line scratch is then introduced into the monolayer to create a wound, followed by the removal of suspended cell debris through washing with PBS. Next, serum‐free or low‐serum medium is added to reduce the impact of cell proliferation on the experimental outcomes. Afterward, the necessary drugs or treatment agents are introduced, and the culture plate is incubated at 37°C with 5% CO_2_. The closure of the scratch is monitored at 0, 24, and 48 h. Finally, the scratch area is quantified using image analysis software.

### 2.8. Transwell Assay

BC cell lines were cultivated in six‐well plates until the requisite confluence was attained. Subsequent to pretreatments, cells were subjected to trypsin digestion and resuspended in a serum‐free medium. Transwell inserts were positioned in 24‐well plates, with 500 *μ*L of complete growth media introduced into the lower chamber and 200 *μ*L of the cell suspension introduced into the top chamber. The setup was incubated for 48 h under ideal conditions to facilitate cell migration or invasion.

Following incubation, cells on the upper membrane surface were carefully detached, and the residual cells on the bottom were fixed with 4% paraformaldehyde for 30 min. The immobilized cells were subjected to staining with 0.1% crystal violet for 30 min and subsequently rinsed to eliminate surplus stain. The stained cells were examined microscopically and quantified to evaluate their invasive or migratory capacity.

### 2.9. Statistical Analysis

To compare the two groups, we employed a *T*‐test, whereas for comparisons involving three or more groups, we utilized one‐way ANOVA. These statistical analyses were carried out using GraphPad Prism software.

### 2.10. Cell Lines

The cell line SVHUC (RRID: CVCL_3798) was used in this study. This cell line was derived from the ureteral epithelial tissue of an 11‐year‐old human male. The cell line T24 (RRID: CVCL_0554) was also used. This cell line was derived from an in situ bladder tumor tissue of an 82‐year‐old human female.

Both cell lines were kind gifts from the laboratory of The First Affiliated Hospital of Harbin Medical University in June 2023. Prior to use, these cell lines were authenticated by short tandem repeat (STR) profiling, and their STR patterns were compared against and confirmed to match the reference profiles in the standard ATCC database. All cells were regularly tested and confirmed to be free of mycoplasma contamination.

## 3. Results

### 3.1. HSPB6 Was Identified as a Signature Gene for BC by Machine Learning Based on the GEO Datasets

Four datasets (GSE13507, GSE3167, GSE52519, and GSE65635) were obtained from the GEO database. Principal component analysis revealed observed significant differences among the datasets (Figure S1A), prompting us to normalize them (Figure S1B) and conduct a differential expression analysis (Figure S1C). GO enrichment analysis indicated that these genes were primarily enriched in cell and extracellular matrix related functions (Figure S1D). KEGG analysis indicated that these genes were primarily related to classical tumor‐associated pathways, including focal adhesion and so on (Figure S1E). GSEA enrichment analysis indicated that these genes were enriched in classical signaling pathways in tumors, such as FOCAL ADHESION, BLADDER CANCER, CELL CYCLE, etc. (Figure S1F,G). Additionally, disease ontology (DO) enrichment analysis revealed that these differentially expressed genes were enriched in renal cell carcinoma and BC (Figure S2A), demonstrating that the screened genes effectively represented BC.

**Figure 1:**
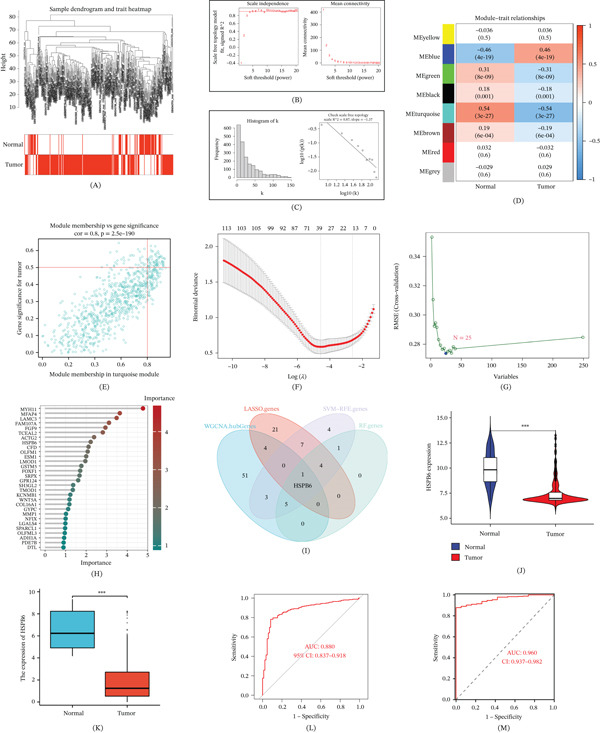
Screening of bladder cancer (BC)–specific genes. (A–C) The process of initial parameter selection in WGCNA. (D, E) The correlation between gene modules, especially turquoise, and BC. (F) LASSO was used to screen specific genes of BC. (G) SVM‐RFE was used to screen specific genes of BC. (H) Random forest was used to screen specific genes of BC. (I) The intersecting gene selected by various methods is HSPB6. (J) Based on the combined GEO dataset, HSPB6 expression was different in normal tissues and BC tissues. (K) Based on the TCGA, HSPB6 expression was different in normal tissues and BC tissues. (L, M) ROC curve of HSPB6 as a diagnostic molecule in GEO and TCGA datasets.  ^∗^ *p* < 0.05,  ^∗∗^ *p* < 0.01, and  ^∗∗∗^ *p* < 0.001.

We utilized WGCNA to develop a gene coexpression network. Initially, we assessed the sample clustering dendrogram of the BC samples after excluding outliers (Figure 1A) and we found that a soft thresholding power of “4” was optimal according to the scale‐independence and average‐connectivity (Figure 1B). This selected power value effectively constructed a scale‐free network, achieving an *R* ^2^ value of 0.87 (Figure 1C).

Subsequently, we classified the BC‐related genes into eight distinct coexpression modules, each represented by a different color. To visualize the correlation analysis results between each module and BC, we generated a heatmap, which revealed that the turquoise exhibited the most tightness (*r* = −0.54, *p* = 3*e* − 27) (Figure 1D). This module was also critical for distinguishing whether a sample was identified as BC (Figure S2B). Ultimately, we identified 64 genes associated with BC from the turquoise module by setting the “module membership” threshold at 0.8 and the “gene significance” threshold at 0.5 (Figure 1E). In conclusion, we successfully identified 64 genes closely associated with BC through the construction of a gene coexpression network.

We employed various methods to refine our search for pivotal BC genes. Initially, the least absolute shrinkage and selection operator (LASSO) screening led to the identification of 37 genes (Figure 1F), followed by the extraction of 17 genes using SVM‐RFE (Figure 1G). The random forest approach revealed an additional 37 genes (Figure 1H). Ultimately, we identified a common gene, HSPB6 (Figure 1I). Therefore, we decided to focus further investigations on HSPB6.

Analysis of the four GEO datasets indicated that HSPB6 was significantly downregulated in most tumors compared with that in normal tissues (Figure 1J). Similarly, TCGA data confirmed the downregulation of HSPB6 in tumors (Figure 1K). Moreover, ROC curve analysis demonstrated that HSPB6 is a highly accurate diagnostic marker for BC (Figure 1L,M).

### 3.2. HSPB6 Is Associated With Poor Prognosis and Poor Clinical Features in BC

**Figure 2:**
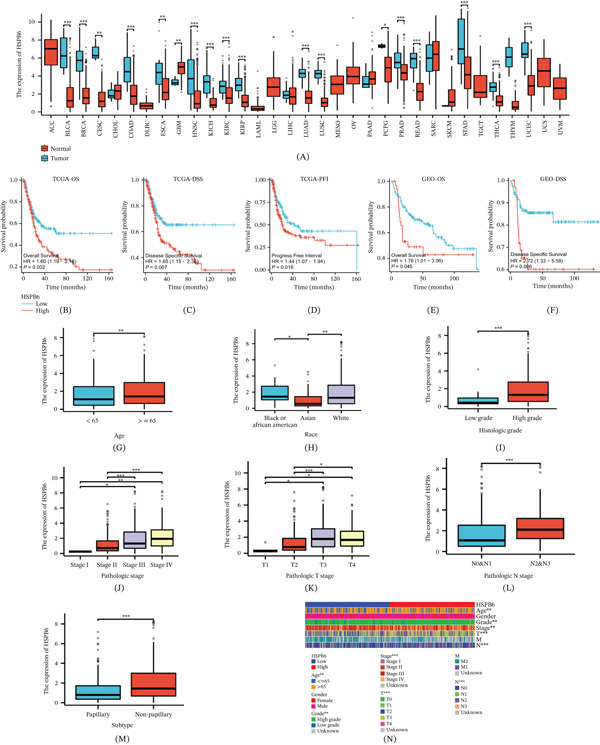
Clinical Significance of HSPB6 in BC. (A) Expression levels of HSPB6 in various tumors. (B–D) Analysis of the relationship between HSPB6 and overall survival (OS), disease‐specific survival (DSS), and progression‐free interval (PFI) based on the TCGA database. (E, F) Analysis of the relationship between HSPB6 and OS combined with DSS based on the GSE13507 dataset. (G–M) Expression differences of HSPB6 across various clinical characteristics. (N) Heatmap showing the correlation between HSPB6 expression levels and various clinical characteristics.  ^∗^ *p* < 0.05,  ^∗∗^ *p* < 0.01, and  ^∗∗∗^ *p* < 0.001.

Initially, we found that HSPB6 was downregulated in most tumors, including BC, breast cancer, and colon cancer, when compared with normal tissues (Figure 2A). Subsequently, we categorized the samples into two groups based on the median expression level of HSPB6: those with high expression and those with low expression. Using clinical data from TCGA along with HSPB6 expression data, we performed survival analyses and generated Kaplan–Meier curves. The results indicated that the group with low HSPB6 expression had significantly better survival outcomes compared with the high‐expression group, regardless of overall survival, disease‐specific survival, or progression‐free interval (Figure 2B–D). Further analyses using clinical data from the GSE13507 dataset corroborated these findings (Figure 2E,F).

Next, we examined the relationship between HSPB6 expression and the clinical features of patients. Differential analysis revealed that HSPB6 expression was significantly higher in individuals aged > 65 years (Figure 2G). Additionally, we observed variations among different ethnic groups, with the lowest expression of HSPB6 observed in Asians, a slightly higher expression in black individuals, and the highest levels in white individuals (Figure 2H).

Furthermore, HSPB6 expression significantly increased with higher tumor grade, stage, T stage, N stage, and nonpapillary tumors (Figure 2I–M). A heat map was constructed to visually represent the specific distributions and differences in clinical features between the HSPB6—high and HSPB6—low expression groups (Figure 2N).

### 3.3. Functional Exploration of HSPB6 in BC

**Figure 3:**
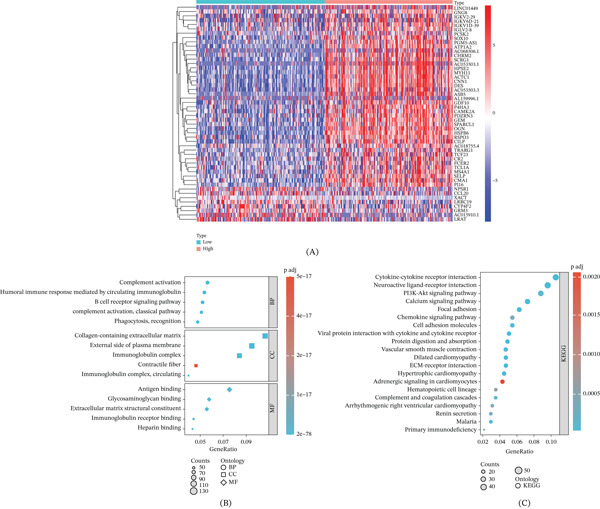
Functional exploration of HSPB6 in BC. (A) Differential genes based on the expression levels of HSPB6 in BC. (B) GO analysis based on these differential genes. (C) KEGG analysis based on these differential genes.

Initially, we identified differentially expressed genes between the Heat Shock Protein Beta‐6—high expression (HHE) and Heat Shock Protein Beta‐6—low expression (HLE) groups (Figure 3A). GO analysis suggested that these genes were related to immunity and extracellular environment (Figure 3B), and KEGG analysis suggested that these genes were closely related to PI3K/Akt (Figure 3C).

### 3.4. Immune Infiltration Analysis of HSPB6 in BC

**Figure 4:**
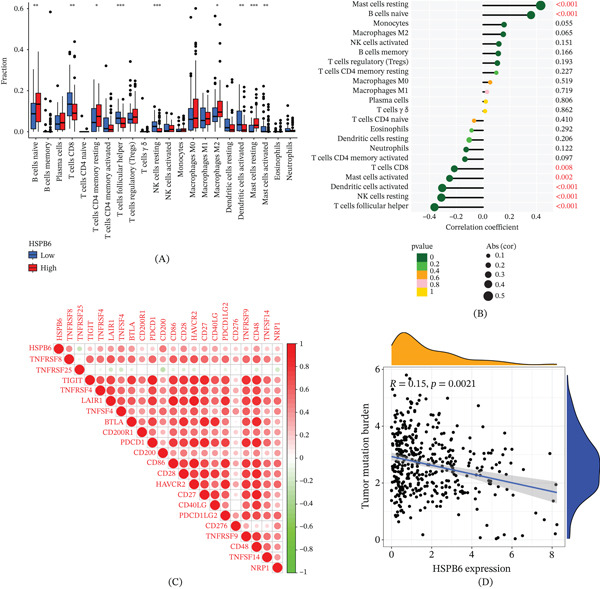
Immune function analysis of HSPB6 in bladder cancer. (A) Differences in immune cell infiltration between the HSPB6 high‐expression group and the HSPB6 low‐expression group. (B) Correlation between HSPB6 expression levels and immune cells. (C) Correlation between HSPB6 expression levels and immune checkpoints. (D) Correlation between HSPB6 expression levels and tumor mutation burden (TMB).  ^∗^ *p* < 0.05,  ^∗∗^ *p* < 0.01, and  ^∗∗∗^ *p* < 0.001.

We analyzed the differences in immune between HHE and HLE groups, aiming to investigate the immunological implications of HSPB6 expression. The infiltration levels of CD8+ T, follicular helper T, resting NK, activated dendritic, and activated mast cells were significantly reduced in the HHE group relative to the HLE group. In contrast, the presence of M2 macrophages, commonly linked to tumor progression and immune suppression, was significantly elevated in the HHE group (Figure 4A).

We performed correlation analyses to clarify the relationship between HSPB6 expression and immune cell infiltration. The findings indicated a significant negative correlation between HSPB6 expression and the infiltration levels of CD8+ T cells, activated mast cells, activated dendritic cells, resting NK cells, and follicular helper T cells (Figure 4B). These observations indicate that increased HSPB6 expression may play a role in creating an immunosuppressive microenvironment by diminishing the presence of essential immune effector cells.

The analysis indicated a positive correlation between HSPB6 expression and various immune checkpoints, such as CD200, TNFSF4, and CD28 (Figure 4C). The observed positive correlation suggests that HSPB6 may affect immune evasion mechanisms via its interaction with these regulatory molecules.

Then, we investigated the relationship between HSPB6 expression and tumor mutation burden (TMB) (Figure 4D), indicating that elevated HSPB6 levels may correlate with a less immunogenic tumor environment.

### 3.5. The Antitumor Effect of HSPB6 Was Verified

**Figure 5:**
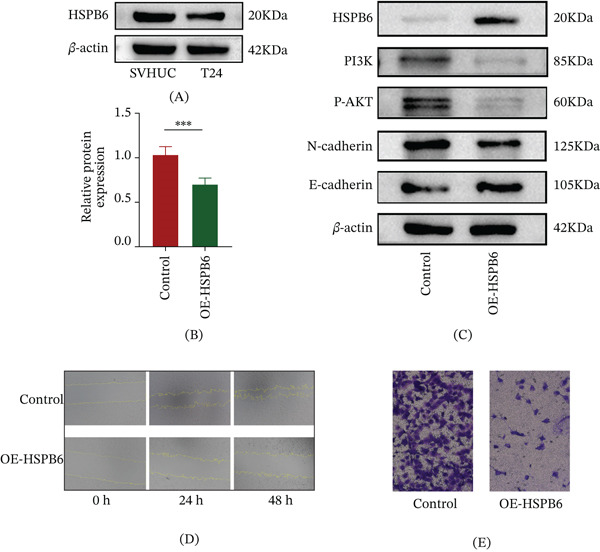
The inhibitory effect of HSPB6 on bladder cancer was verified. (A) Expression differences of HSPB6 between normal cell lines and bladder cancer cell lines. (B) CCK‐8 assay after overexpression of HSPB6. (C) Changes in the expression of related proteins in the bladder cancer cell line T24 after overexpression of HSPB6. (D) Wound healing assay after overexpression of HSPB6. (E) Transwell assay after overexpression of HSPB6.  ^∗^ *p* < 0.05,  ^∗∗^ *p* < 0.01, and  ^∗∗∗^ *p* < 0.001.

We assessed HSPB6 expression in the SVHUC and the BC cell Line T24 (Figure 5A) about the HSPB6. The results indicated that the expression level of HSPB6 in tumor tissues was lower than that in normal tissues, and this finding was also confirmed by the immunohistochemical data from The Human Protein Atlas database (Figure S3A,B). To investigate the functional role of HSPB6, we overexpressed it in the T24 using overexpression plasmids. The subsequent CCK‐8 assay revealed that HSPB6 overexpression significantly inhibited the proliferation of T24 cells (Figure 5B). The results showed that overexpressing HSPB6 suppressed the expression of key proteins in the PI3K/Akt signaling pathway, specifically PI3K and P‐AKT, which are critical for promoting tumor growth and survival. Additionally, HSPB6 overexpression led to decreased expression of N‐cadherin, while increasing the expression of E‐cadherin (Figure 5C).

These findings collectively suggest that HSPB6 inhibits tumor cell proliferation and migration by modulating the PI3K/Akt signaling pathway and EMT‐related proteins, positioning it as a promising candidate for therapeutic intervention in BC.

Additionally, wound healing and Transwell assays confirmed that HSPB6 overexpression inhibited the migration of T24 cells (Figure 5D,E).

**Figure 6:**
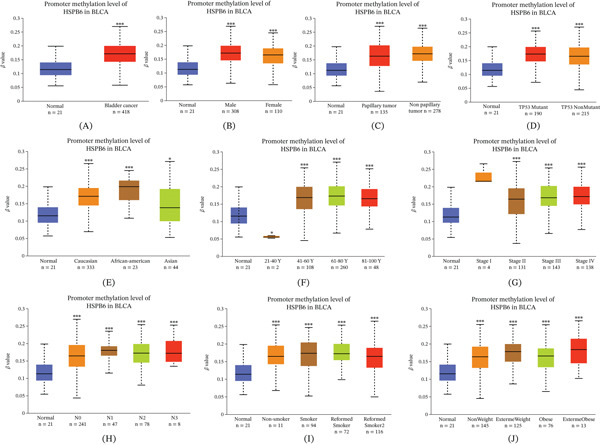
Methylation levels of HSPB6 in bladder cancer. (A) The methylation level of HSPB6 in bladder cancer tissues was significantly higher than that in normal tissues. (B–J) The methylation level of HSPB6 in all clinical subgroups of bladder cancer was significantly higher than that in normal tissues.  ^∗^ *p* < 0.05,  ^∗∗^ *p* < 0.01, and  ^∗∗∗^ *p* < 0.001.

We utilized UALCAN to analyze the methylation levels of HSPB6. Our analysis indicated that the methylation level of HSPB6 was significantly elevated in BC patients compared with normal tissue. Furthermore, we observed that HSPB6 methylation levels were significantly higher across various clinical subgroups, including sex, pathological classification, TP53 mutation status, ethnicity, age, cancer stage, N stage, smoking status, and body weight status when compared with normal tissue samples (Figure 6A–J).

### 3.6. The Prognostic Model Was Constructed Based on HSPB6

**Figure 7:**
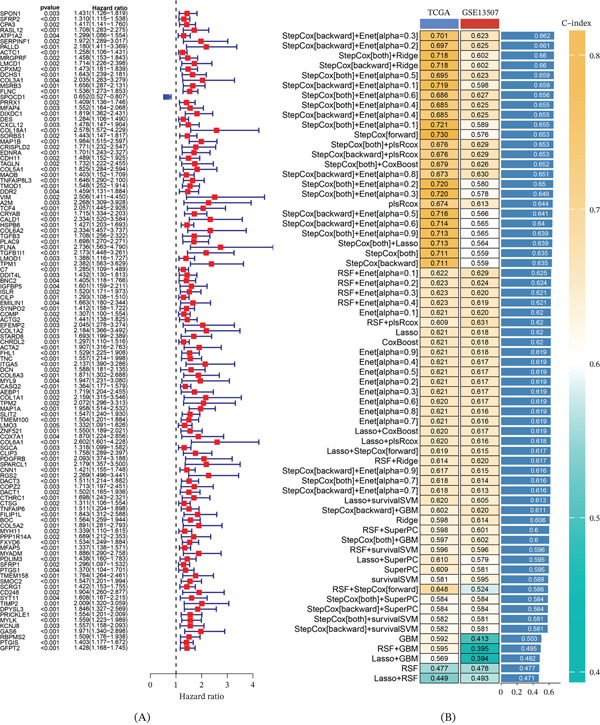
Construction of the prognostic model. (A) A total of 115 survival genes were identified by Cox regression analysis. (B) Multiple prognostic models were constructed by combining multiple machine learning methods and ranked from largest to smallest AUC values, and StepCox[backward] + Enet[alpha = 0.3] has the highest AUC value.

Subsequently, we utilized the GSE13507 dataset to categorize samples into HSPB6 high‐expression and low‐expression groups based on the median expression level of HSPB6. Through differential gene analysis, we identified 367 genes, and by performing intersection analysis, we recognized 196 commonly differentially expressed genes from these two independent datasets (Figure S4A). To further investigate the correlation between these genes and patient survival, we conducted a Cox regression analysis, which identified 115 genes significantly associated with survival (Figure 7A).

**Figure 8:**
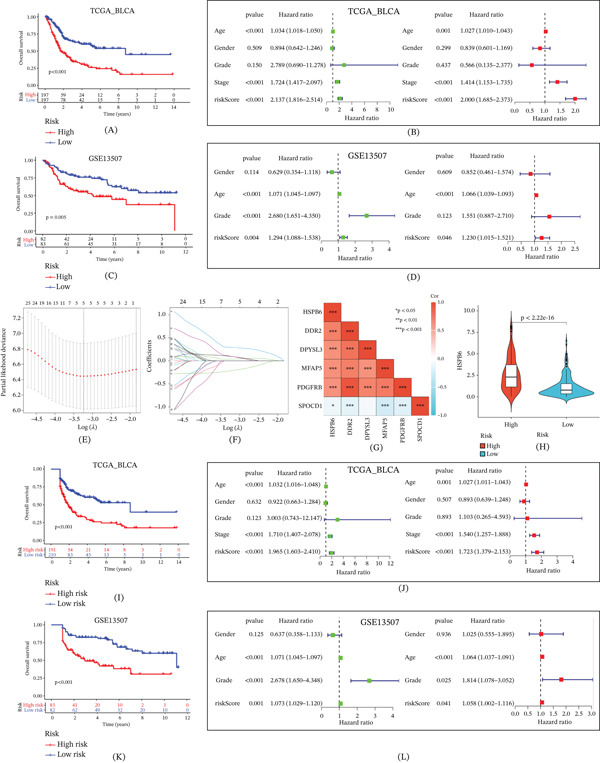
Validation and further analysis of prognostic model. (A) Suvival analysis of A 33‐gene model constructed by StepCox[backward] + Enet[alpha = 0.3] based on the TCGA. (B) Univariate and multivariate analysis proved that the model could independently predict the prognosis of patients which from TCGA. (C, D) The same verification was obtained in GSE13507. (E, F) Further screening of the 33 genes was conducted using LASSO Cox regression analysis. (G) Correlation between HSPB6 and the genes constituting the risk model. (H) Expression differences of HSPB6 between high‐risk and low‐risk groups. (I) Suvival analysis of the prognostic model which contain the DDR2, DPYSL3, MFAP5, PDGFRB, and SPOCD1. (J) Univariate and multivariate analysis proved that the model could independently predict the prognosis of patients which from TCGA. (K, L) The same verification was obtained in GSE13507.  ^∗^ *p* < 0.05,  ^∗∗^ *p* < 0.01, and  ^∗∗∗^ *p* < 0.001.

Building on this foundation, we employed a variety of machine learning algorithms, including XGBoost, ridge regression, SVM, random survival forests, and LASSO, resulting in a total of 101 different combinations to construct prognostic models. By comparing the performance of these models, we found that the model constructed using the StepCox[backward] combined with the Enet[alpha = 0.3] algorithm exhibited the highest AUC value, incorporating 33 genes (Figure 7B). Survival analysis conducted using the TCGA dataset revealed a clear and statistically significant prognostic distinction between the low‐risk and high‐risk groups. Patients classified in the low‐risk group demonstrated markedly better survival outcomes compared with those in the high‐risk group. The robustness of the model was reinforced by univariate and multivariate Cox regression analyses, which identified risk scores as an independent prognostic factor. These findings suggest that the model′s predictive capability is not confounded by other variables and that it reliably contributes unique prognostic value (Figure 8A,B). Furthermore, the analysis results from the GSE13507 dataset supported this finding (Figure 8C,D).

To enhance the efficiency and practicality of the prognostic model, we implemented a refinement process to optimize its construction. This involved applying LASSO Cox regression analysis, systematically narrowing down the list by penalizing less significant genes, retaining only those with the strongest prognostic associations. This process ensures that the final model is not only more streamlined but also retains its predictive accuracy and robustness. Ultimately, we established a streamlined prognostic model composed of five genes: DDR2, DPYSL3, MFAP5, PDGFRB, and SPOCD1 (Figure 8E,F). In this model, HSPB6 was positively correlated with DDR2, DPYSL3, MFAP5, PDGFRB, and negatively correlated with SPOCD1 (Figure 8G). The developed risk model effectively stratified patients with BC into high‐risk and low‐risk groups, providing critical insights into prognosis. Notably, the HSPB6 was higher in the high‐risk group (Figure 8H), highlighting a potential relationship between HSPB6 expression and patient risk profiles. Patients in the low‐risk group exhibited significantly better survival rates than those in the high‐risk group (Figure 8I). These results underscore the model′s ability to reflect survival probabilities with high accuracy, offering valuable prognostic insights. Moreover, univariate and multivariate Cox regression analyses confirmed that the risk scores derived from the model serve as an independent prognostic factor. This finding was consistent across multiple datasets, including the TCGA dataset (Figure 8J) and the GSE13507 dataset (Figure 8K,L), reinforcing the robustness and generalizability of the model.

**Figure 9:**
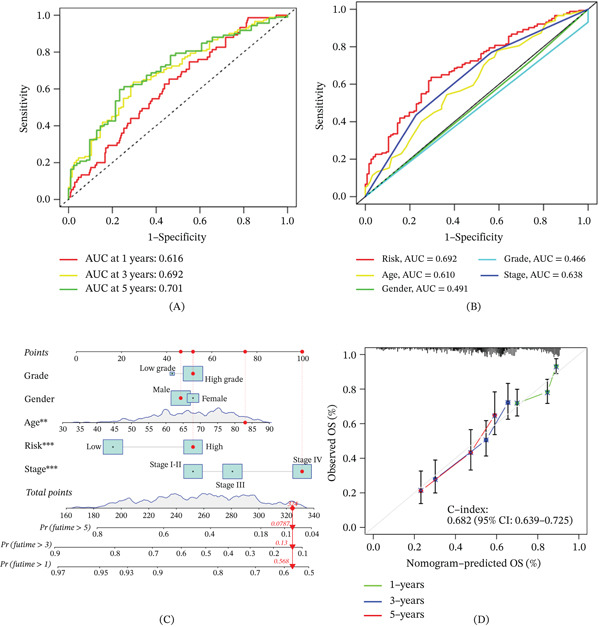
Evaluation of prognostic model and nomogram construction. (A) The ROC curve proved that the prognostic model had superior predictive performance in predicting patient survival. (B) ROC curve proved that the predictive performance of the model was better than other clinical features. (C) A nomogram was constructed based on clinical features and prognostic models. (D) The C‐index curve proves that the nomogram map has better prediction performance.  ^∗^ *p* < 0.05,  ^∗∗^ *p* < 0.01, and  ^∗∗∗^ *p* < 0.001.

**Figure 10:**
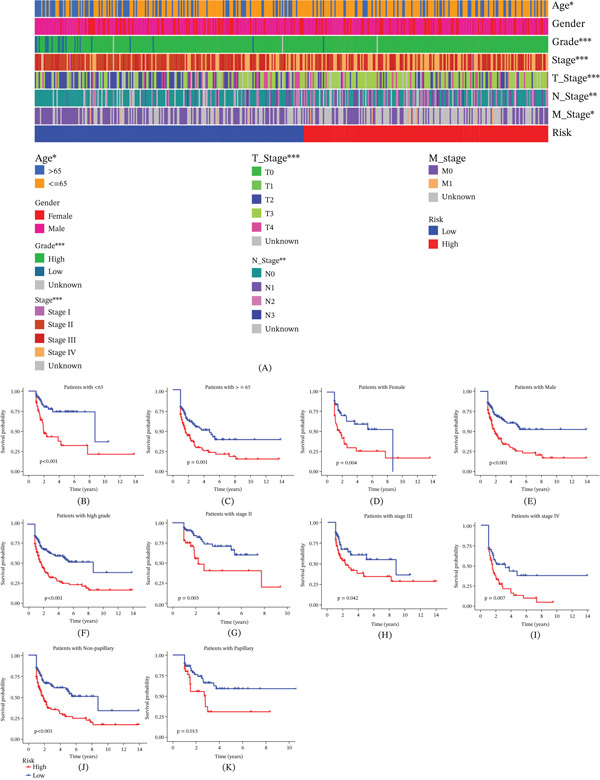
Clinical significance of prognostic model in BC. (A) Differences in clinical features between high‐ and low‐risk groups. (B–K) The prognostic model can accurately predict patient outcomes in various subgroups of clinical characteristics.  ^∗^ *p* < 0.05,  ^∗∗^ *p* < 0.01, and  ^∗∗∗^ *p* < 0.001.

ROC analysis demonstrated that the model effectively predicted patient survival with higher AUC (Figure 9A). The proposed risk model demonstrated superior predictive performance compared with traditional clinical features (Figure 9B). To further refine the prognostic capabilities of this model, a nomogram was developed, integrating key clinical factors—age, sex, tumor stage, and tumor grade—along with the risk model itself (Figure 9C) and this nomogram enables the prediction of survival rates for patients with BC efficiently (Figure 9D). The clinical manifestations of patients in the high‐risk group and low‐risk group were significantly different (Figure 10A), and this risk model effectively predicts patient prognosis across all clinical subgroups (Figure 10B–K).

Furthermore, the GO indicated enrichment in functions related to the extracellular matrix, such as structural constituents, tensile strength–conferring constituents, and extracellular matrix binding (Figure S5A). KEGG analysis highlighted enrichment in classical tumor pathways, particularly the PI3K/Akt signaling pathway (Figure S5B). GSEA revealed the similarly results (Figure S5C,D).

In the final stage of our analysis, we examined the expression levels of immune checkpoints in patients stratified into high‐risk and low‐risk groups based on our risk model. Our results revealed that the expression of immune checkpoint molecules was significantly elevated in the high‐risk group compared with the low‐risk group, as illustrated in Figure S6A. By highlighting these disparities, our study not only validates the risk stratification model but also provides a basis for personalized immunotherapy approaches tailored to patients’ risk profiles.

## 4. Discussion

BC is one of the most common malignant tumors worldwide, with a particularly high incidence in men. Despite significant advancements in early diagnosis and treatment techniques in recent years, such as the use of cystoscopy and urine cytology for early screening, the recurrence rate of BC remains high at approximately 50%–70%. Current treatment options include surgical resection, chemotherapy, immunotherapy, and targeted therapy. However, these modalities face challenges such as drug resistance, suboptimal efficacy, and adverse effects, particularly in patients with muscle‐invasive BC [32]. Therefore, the identification of novel biomarkers and targeted therapeutic strategies is crucial for improving patient prognosis. Machine learning is increasingly widely applied in gene screening [33]. Therefore, we selected multiple machine learning–based options and ultimately screened out the gene with the strongest specificity: HSPB6.

The HSP family plays a vital role in cellular stress responses, assisting cells to cope with various forms of stress, including heat, oxidative stress, and toxic substances [31, 34]. The HSPB family consists of multiple members, among which HSPB6 is a small HSP that exerts inhibitory effects on various tumor types. Previous studies have indicated that HSPB6 functions by inhibiting cell proliferation and promoting apoptosis in breast, lung, and colorectal cancers. In the context of BC, the specific role of HSPB6 requires further investigation. However, its potential tumor‐suppressive function provides a foundation for future research.

In our research, both public database analysis and protein determination demonstrated that the expression level of HSPB6 in tumors was significantly lower than that in normal tissues. Subsequently, through function acquisition experiment, it was proved that HSPB6 could effectively inhibit the EMT process of BC, but this conflicted with the results of bioinformatics analysis. Firstly, the experimental results are the most reliable, so the anticancer effect of HSPB6 can be determined. Secondly, the core of bioinformatics analysis is to conduct statistical analysis on the relationship between the gene expression levels in clinical samples and the clinical traits of patients. However, this correlation can be interfered with by various confounding factors and does not directly equate to the causal relationship where genes determine prognosis, or as the tumor progresses, HSPB6 may increase compensatorily, thus mistakenly generating highly expressed HSPB6 which is associated with adverse clinical traits. These are all possible reasons. Of course, our research is also limited by cell experiments and lacks in vivo experiments. We believe that further clarification can be achieved in subsequent studies. An increasing number of studies have shown that certain genes play a crucial role in the immune microenvironment of specific tumors [35, 36]. CD8+ T cells are the key to antitumor immunity. After being stimulated by tumor antigens, they are activated and chemotactic to the core area of tumors through various cytokines. They directly kill cancer cells by secreting IFN‐*γ*. Meanwhile, the coinfiltration of CD8+ T cells and NK cells can synergistically destroy tumors. In addition, a high TMB can enhance its infiltration. Our analysis indicates that the expression level of HSPB6 is negatively correlated with the infiltration of CD8+ T cells and NK T cells, but positively correlated with the infiltration of B cells. However, these findings are limited to those based on public databases. Further experimental verification is still needed to draw a clear conclusion.

Additionally, we constructed a high‐performance clinical prognostic model based on HSPB6, which comprises DDR2, DPYSL3, MFAP5, PDGFRB, and SPOCD1. DDR2 is a contributing factor to the development of PD‐1 resistance in BC cells and is involved in the construction of several prognostic models [37–39]; DPYSL3 is significantly overexpressed in BC and correlates with unfavorable patient prognosis [40]; MFAP5 promotes the migration of BC cells [41]; the expression level of PDGFRB, which is associated with copper‐induced cell death, is significantly upregulated in BC and has been shown to participate in the construction of multiple prognostic models [42]; SPOCD1 has been demonstrated to be involved in necroptosis and autophagy processes in BC [43, 44]. All of the above indicate that the several genes involved in the model play important roles in BC.

In summary, the role of HSPB6 in BC warrants in‐depth investigation, as its mechanism of inhibiting EMT through the regulation of the PI3K/Akt signaling pathway provides potential targets for novel therapeutic strategies. Future research should concentrate on the relationship between HSPB6 expression and the tumor microenvironment, as well as on the function of HSPB6 at different stages of tumor progression, with the aim of elucidating its dual role in BC progression and advancing its clinical application. This study not only enriches our understanding of HSPB6 but also opens new avenues for the treatment of BC.

## 5. Conclusions

This work employed data from the GEO and TCGA databases to identify HSPB6 as a pivotal molecule using an integrated bioinformatics methodology. This dual‐database approach facilitated comprehensive analysis, guaranteeing that the research outcomes are statistically valid and physiologically meaningful. Subsequent cellular tests further validated the essential function of HSPB6 in the advancement of BC. The overexpression of HSPB6 was observed to inhibit the PI3K/Akt signaling pathway, thereby lowering EMT, decreasing N‐cadherin levels, and elevating E‐cadherin levels. In light of these findings, HSPB6 presents itself as a viable choice for the diagnostic and treatment methods of BC. Its capacity to modulate critical pathways and molecular indicators associated with cancer advancement establishes it as a significant area for forthcoming study and therapeutic applications, with the potential to reduce BC metastasis and enhance patient outcomes.

**Nomenclature**
BC: bladder cancer
EMT: epithelial–mesenchymal transition
WGCNA: weighted gene coexpression network analysis
LASSO: least absolute shrinkage and selection operator
SVM‐RFE: support vector machine recursive feature elimination
HHE: HSPB6—high expression
HLE: HSPB6—low expression
TCGA: The Cancer Genome Atlas
GEO: Gene Expression Omnibus
GO: Gene Ontology
KEGG: Kyoto Encyclopedia of Genes and Genomes
GSEA: gene set enrichment analysis
OS: overall survival
PFS: progression‐free survival
PFI: progression free interval
sHSPs: small heat shock proteins

## Author Contributions

**Jian-she Wang:** conceptualization, supervision, formal analysis, investigation, and writing—original draft. **Yi-fan Qiu:** software, writing—review and editing, and data curation. **Lu Zhang:** writing—review and editing, data curation, and methodology. **Bo Ji:** writing—review and editing, and methodology. **Sen Liang:** software, writing—review and editing, and data curation. **Ya-Xuan Wang:** software, writing—review and editing, and data curation. **Hai-xia Zhu:** software, writing—review and editing, and data curation. Jian‐she Wang, Yi‐fan Qiu, and Lu Zhang are the co‐first authors.

## Funding

No funding was received for this manuscript.

## Ethics Statement

The authors have nothing to report.

## Conflicts of Interest

The authors declare no conflicts of interest.

## Supporting Information

Additional supporting information can be found online in the Supporting Information section.

## Supporting information


**Supporting Information 1** Figure S1: Obtained bladder cancer data from the GEO database. (A) Distribution of the raw data from four datasets: GSE13507, GSE3167, GSE52519, and GSE65635. (B) Distribution of the merged and normalized data from the four datasets: GSE13507, GSE3167, GSE52519, and GSE65635. (C) Differential genes between normal tissues and bladder cancer tissues in the merged dataset. (D) GO enrichment analysis of the differential genes. (E) KEGG enrichment analysis of the differential genes. (F, G). GSEA enrichment analysis of the differential genes in normal tissues and tumor tissues.

**Supporting Information 2** Figure S2: (A) Differential gene–based disease ontology (DO) enrichment analysis. (B) Gene importance in each module.

**Supporting Information 3** Figure S3: (A) The immunohistochemical data of normal tissue from The Human Protein Atlas. (B) The immunohistochemical data of tumor from The Human Protein Atlas.

**Supporting Information 4** Figure S4: (A) One hundred and ninety‐six HSPB6‐based differential genes were selected in TCGA and GEO.

**Supporting Information 5** Figure S5: (A, B) GO and KEGG enrichment analysis of differentially expressed genes between high‐risk and low‐risk groups. (C, D) GSEA enrichment analysis based on the genes in high‐risk and low‐risk groups.

**Supporting Information 6** Figure S6. (A) Differences in the expression levels of immune checkpoints between high and low risk groups.

**Supporting Information 7** Table S1: The clinical data of TCGA_BLCA.

**Supporting Information 8** Table S2: The clinical data of GSE13507.

## Data Availability

The data generated in this study are publicly available in GEO (https://www.ncbi.nlm.nih.gov/geo/) at GSE13507, GSE3167, GSE52519, and GSE65635, and in TCGA (https://www.ncbi.nlm.nih.gov/) for BC data. Methylation data for HSPB6 were obtained from the online database UALCAN (https://ualcan.path.uab.edu/index.html).
