# Supplementary figures and images for: HSPB6: A Potential Prognostic Biomarker, Inhibiting the Epithelial–Mesenchymal Transition (EMT) Process Through the PI3K/Akt Signaling Pathway Based on the Machine Learning and Experimental Validation
Source: Hum Mutat. 2026 Apr 20;2026:4843618. doi: 10.1155/humu/4843618 (PMC13092927; doi:10.1155/humu/4843618)

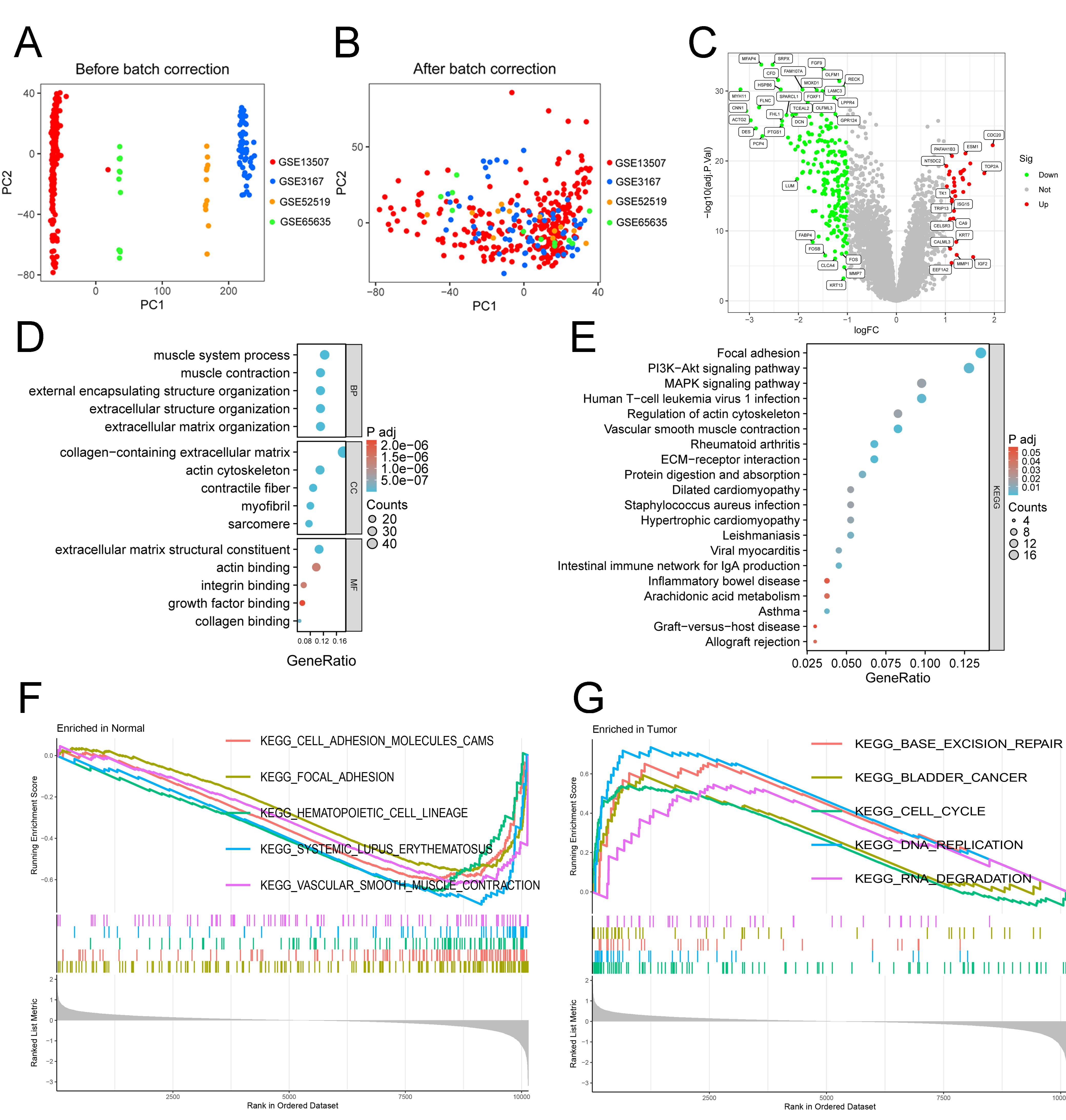

Supplement: Supplementary file 1 — Supporting Information 1 Figure S1: Obtained bladder cancer data from the GEO database. (A) Distribution of the raw data from four datasets: GSE13507, GSE3167, GSE52519, and GSE65635. (B) Distribution of the merged and normalized data from the four datasets: GSE13507, GSE3167, GSE52519, and GSE65635. (C) Differential genes between normal tissues and bladder cancer tissues in the merged dataset. (D) GO enrichment analysis of the differential genes. (E) KEGG enrichment analysis of the differential genes. (F, G). GSEA enrichment analysis of the differential genes in normal tissues and tumor tissues. [file HUMU-2026-4843618-s007.tif]

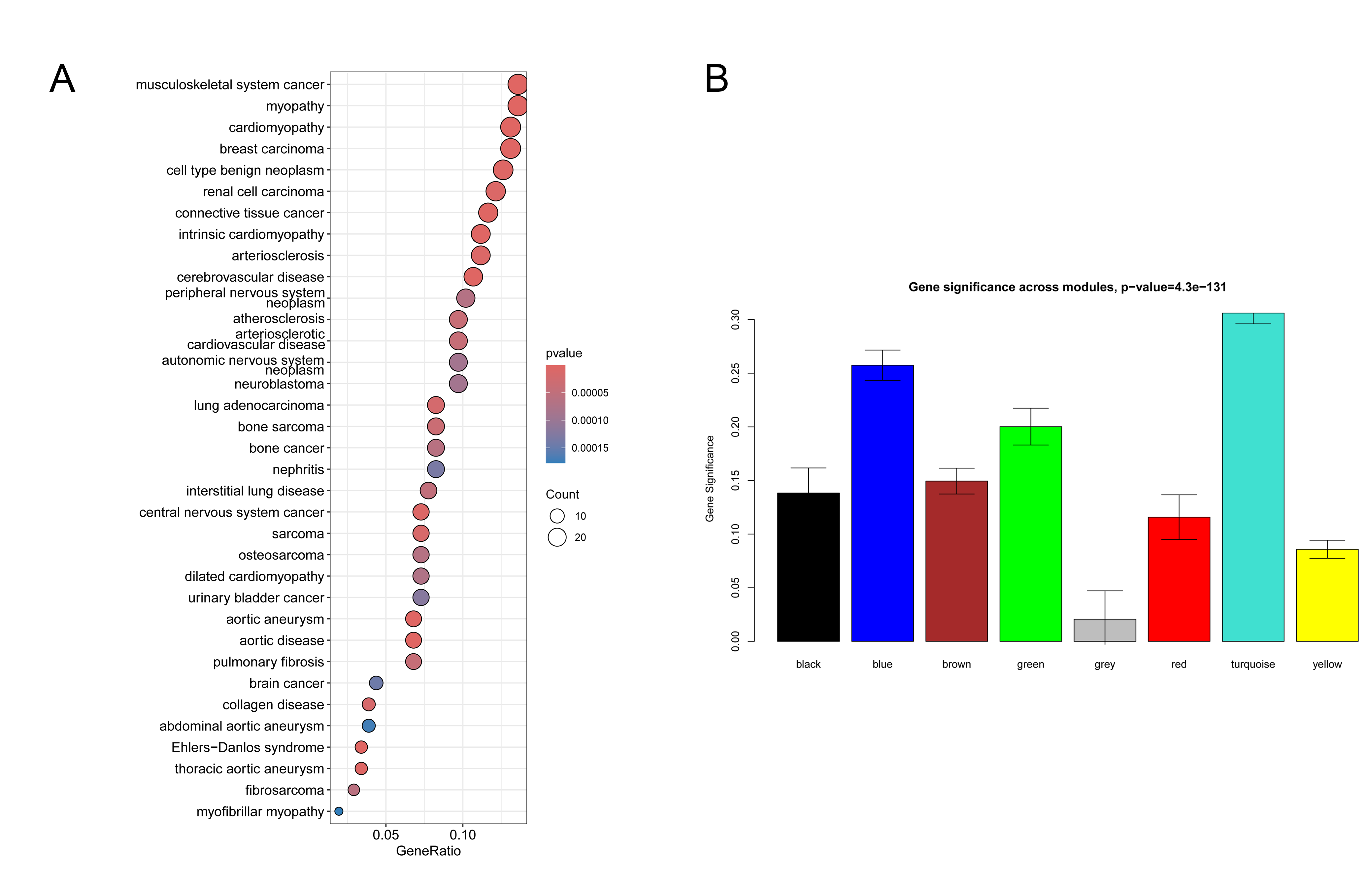

Supplement: Supplementary file 2 — Supporting Information 2 Figure S2: (A) Differential gene–based disease ontology (DO) enrichment analysis. (B) Gene importance in each module. [file HUMU-2026-4843618-s001.tif]

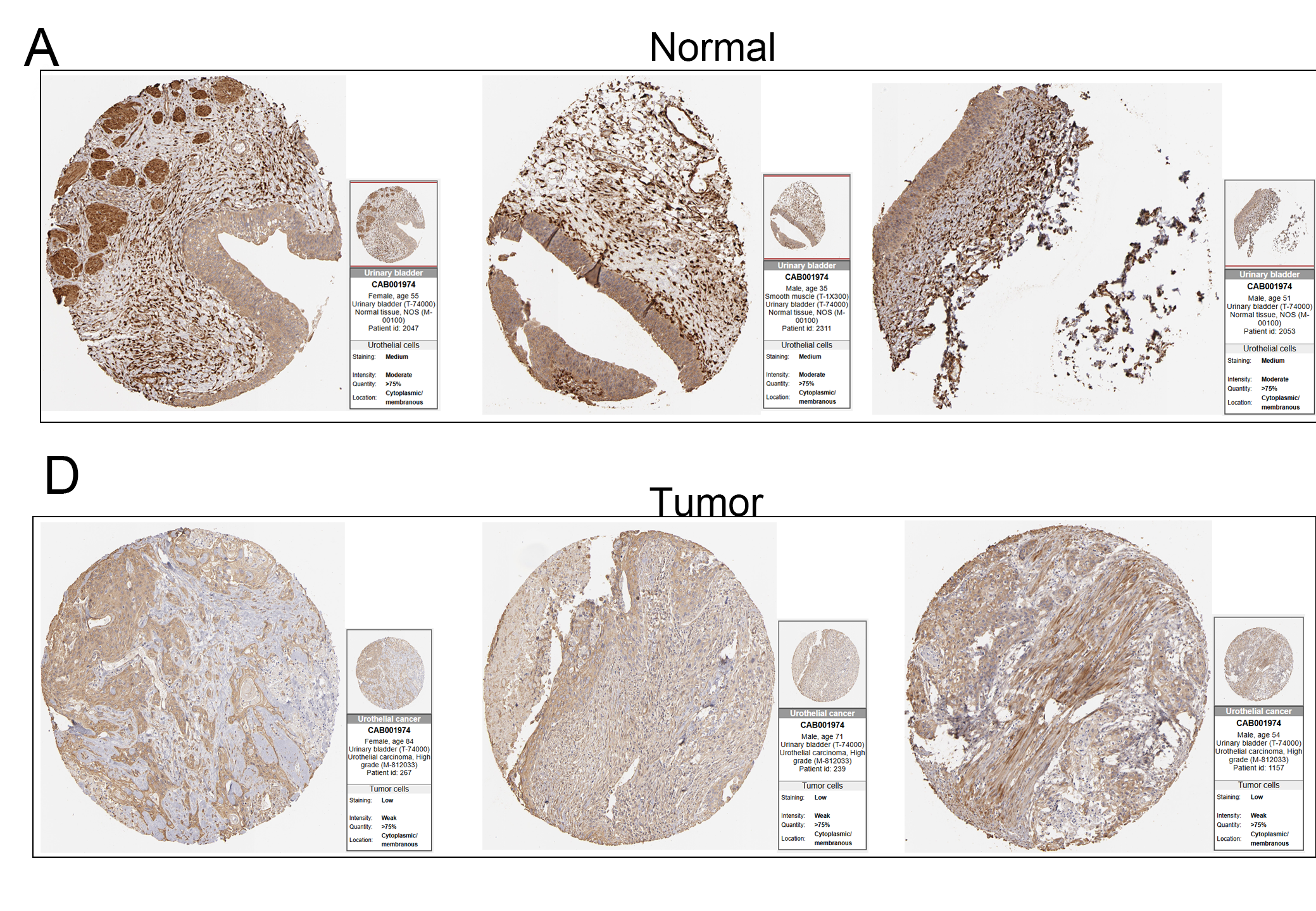

Supplement: Supplementary file 3 — Supporting Information 3 Figure S3: (A) The immunohistochemical data of normal tissue from The Human Protein Atlas. (B) The immunohistochemical data of tumor from The Human Protein Atlas. [file HUMU-2026-4843618-s002.tif]

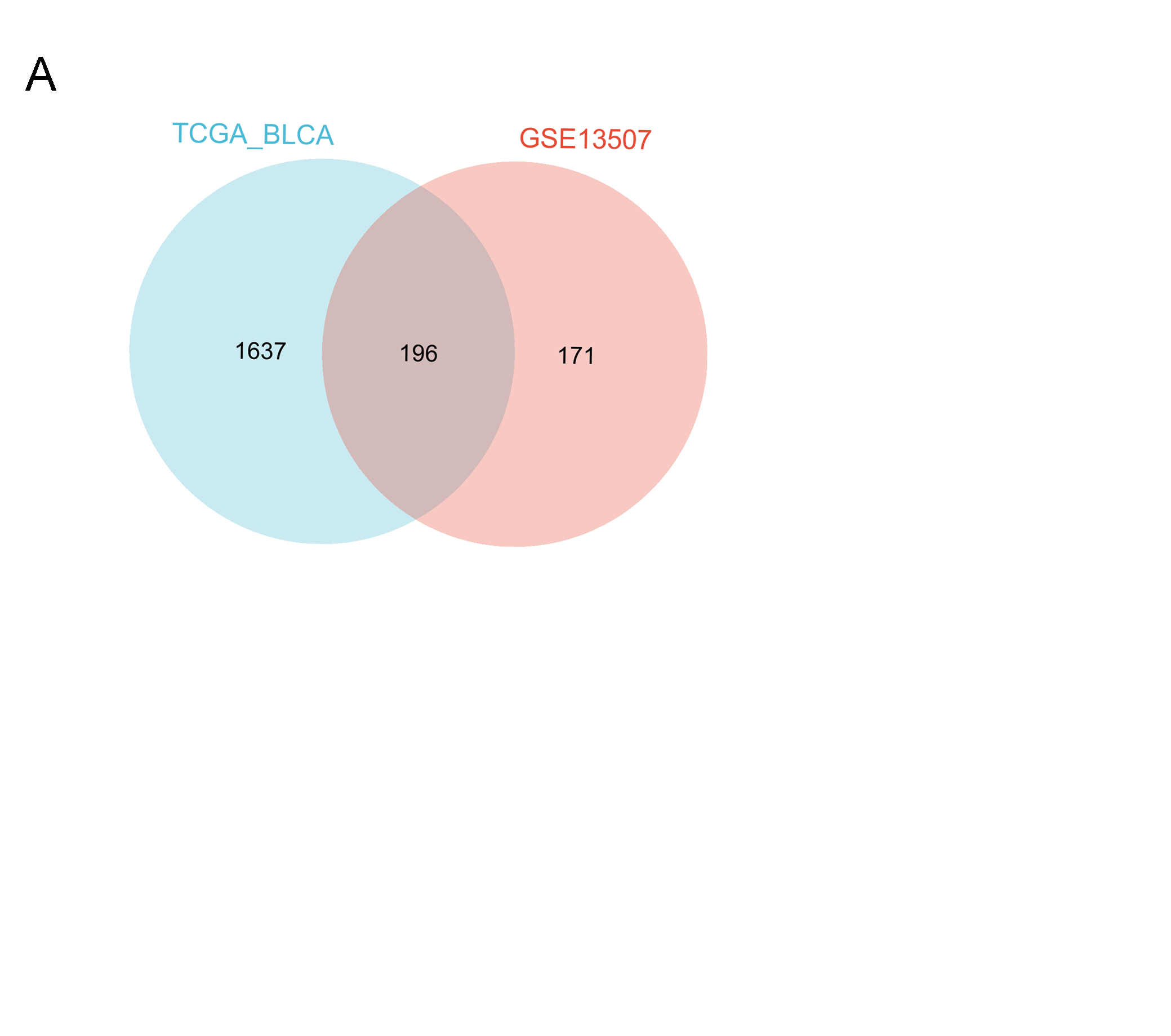

Supplement: Supplementary file 4 — Supporting Information 4 Figure S4: (A) One hundred and ninety‐six HSPB6‐based differential genes were selected in TCGA and GEO. [file HUMU-2026-4843618-s008.tif]

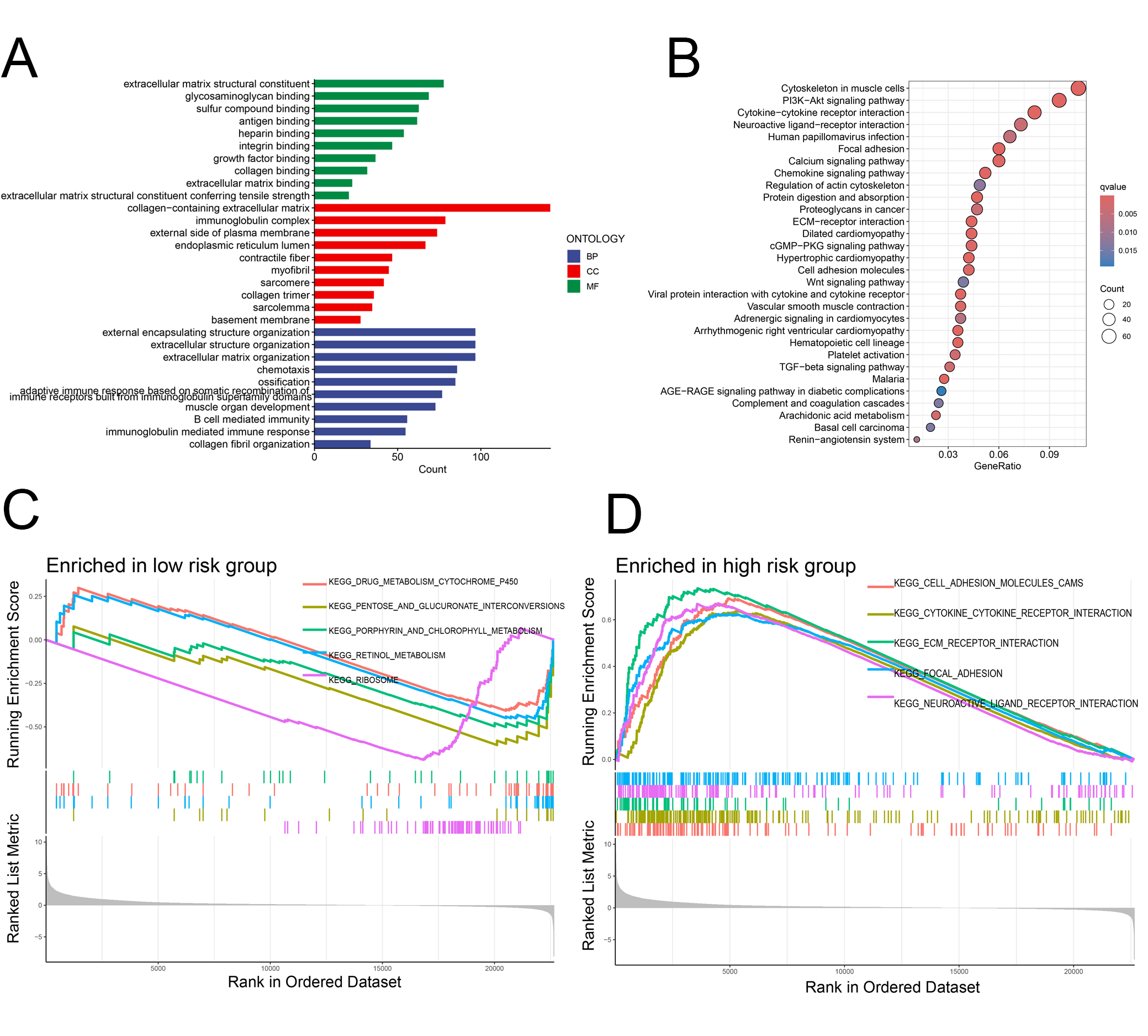

Supplement: Supplementary file 5 — Supporting Information 5 Figure S5: (A, B) GO and KEGG enrichment analysis of differentially expressed genes between high‐risk and low‐risk groups. (C, D) GSEA enrichment analysis based on the genes in high‐risk and low‐risk groups. [file HUMU-2026-4843618-s004.tif]

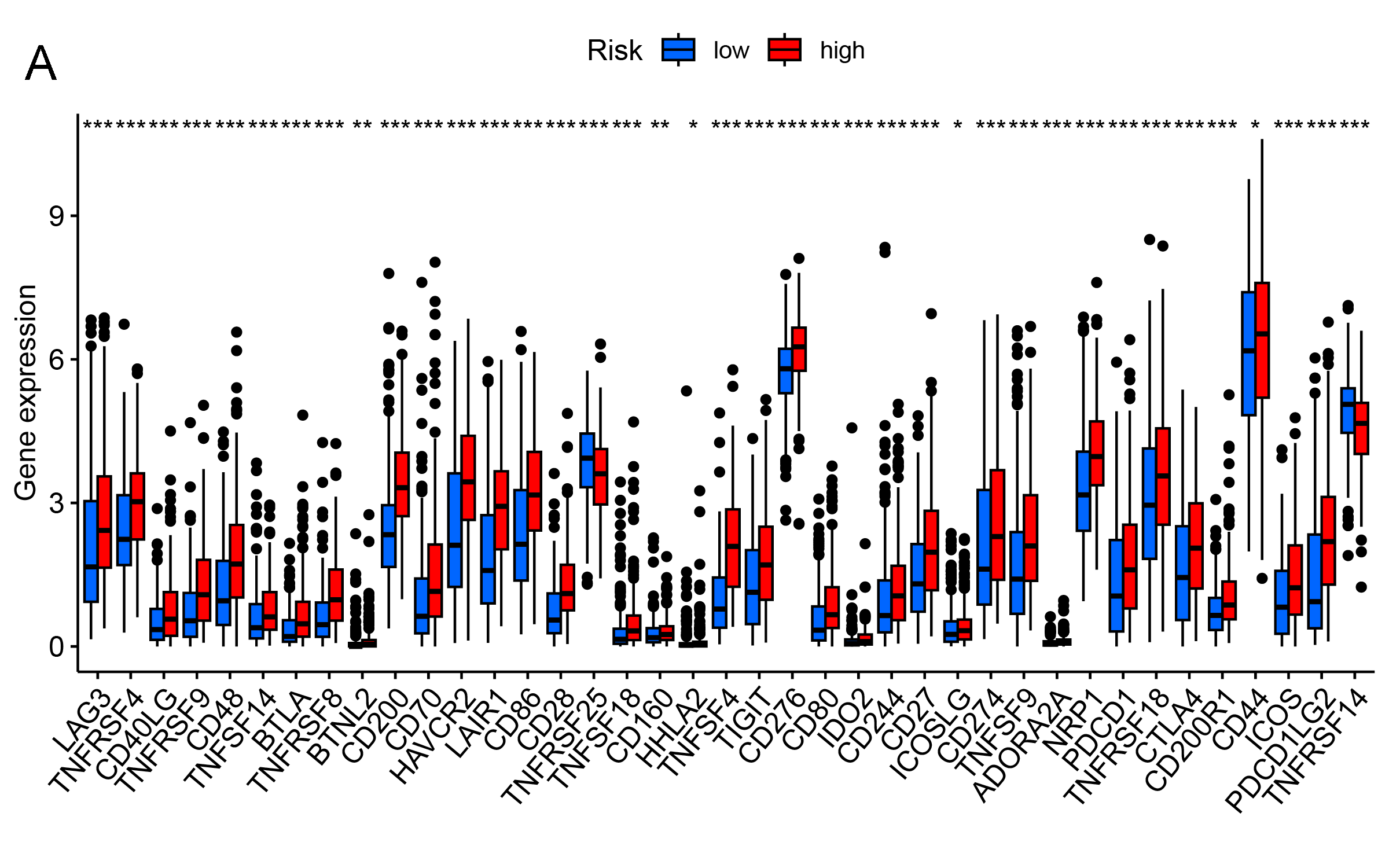

Supplement: Supplementary file 6 — Supporting Information 6 Figure S6. (A) Differences in the expression levels of immune checkpoints between high and low risk groups. [file HUMU-2026-4843618-s005.tif]
